# Genomic and non-genomic effects of androgens in the cardiovascular system: clinical implications

**DOI:** 10.1042/CS20170090

**Published:** 2017-06-23

**Authors:** Angela K. Lucas-Herald, Rheure Alves-Lopes, Augusto C. Montezano, S. Faisal Ahmed, Rhian M. Touyz

**Affiliations:** 1 Developmental Endocrinology Research Group, Queen Elizabeth University Hospital Campus, 1345 Govan Road, Glasgow G51 4TF, U.K.; 2 Institute of Cardiovascular and Medical Sciences, British Heart Foundation Glasgow Cardiovascular Research Centre, University of Glasgow, 126 University Place, Glasgow G12 8TA, U.K.

**Keywords:** androgen receptor, cardiovascular, genomic, non-genomic

## Abstract

The principle steroidal androgens are testosterone and its metabolite 5α-dihydrotestosterone (DHT), which is converted from testosterone by the enzyme 5α-reductase. Through the classic pathway with androgens crossing the plasma membrane and binding to the androgen receptor (AR) or via mechanisms independent of the ligand-dependent transactivation function of nuclear receptors, testosterone induces genomic and non-genomic effects respectively. AR is widely distributed in several tissues, including vascular endothelial and smooth muscle cells. Androgens are essential for many developmental and physiological processes, especially in male reproductive tissues. It is now clear that androgens have multiple actions besides sex differentiation and sexual maturation and that many physiological systems are influenced by androgens, including regulation of cardiovascular function [nitric oxide (NO) release, Ca^2+^ mobilization, vascular apoptosis, hypertrophy, calcification, senescence and reactive oxygen species (ROS) generation]. This review focuses on evidence indicating that interplay between genomic and non-genomic actions of testosterone may influence cardiovascular function.

## Clinical perspectives

• Androgens have many extragonadal effects, including regulation of the cardiovascular system.
• Via activation of genomic and non-genomic signalling pathways, androgens can lead to divergent outcomes.
• In the vascular system, androgens have the potential to cause NO release, Ca^2+^ mobilization, vascular apoptosis, hypertrophy, calcification, senescence and ROS generation.

## Introduction

Steroid hormones, including androgens, are involved in many developmental and physiological processes [1–4]. Classically androgens mediate their effects via binding to the androgen receptor (AR), a ligand-dependent transcription factor and a member of the nuclear receptor gene superfamily [5,6]. Additionally, androgens can activate signalling pathways via non-DNA binding-dependent actions [7,8]. AR is expressed in many tissues, with the highest concentration being in the male reproductive organs [9]. The *AR* has three functional domains: exon 1 encodes the N-terminal transactivation domain (NBD), exons 2 and 3 encode the DNA-binding domain (DBD) and exons 4–8 encode the C-terminus ligand-binding domain (LBD) [10,11].

The AR is expressed in many cells of the cardiovascular system including cardiomyocytes [12], endothelial cells [13], vascular smooth muscle cells (VSMCs) [14], fibroblasts [15], macrophages [16] and platelets [17]. There are sex differences in AR expression; males have significantly higher levels of AR mRNA than females [18]. In addition, males demonstrate greater AR activation with endogenous testosterone, and prolonged exposure to endogenous testosterone leads to up-regulation of AR [19].

Testosterone is the principal male steroid hormone from the androgen family. Approximately 95% of endogenous testosterone is produced by the testes and it is secreted by the Leydig cells [20]. Small amounts of testosterone are also secreted by the zona reticularis of the adrenal glands [21]. The physiological levels of testosterone in men range from 10 to 30 nM with lower levels found in women (0.6–2.5 nM) [22]. Testosterone is synthesized from cholesterol and stored in lipid droplets through a series of reactions that occur in mitochondria and microsomes (smooth endoplasmic reticulum and surrounding cytoplasm). The first step of steroidogenesis is a transfer of cholesterol to the internal mitochondrial membrane with subsequent bioconversion in pregnenolone by the enzyme cytochrome P450 SCC [23]. Pregnenolone is then transferred to the endoplasmic reticulum where it is converted into testosterone through a series of enzymatic reactions via generation of 17α-hydroxypregnenolone and subsequent formation of dehydroepiandrosterone (DHEA) or via 17α-hydroxyprogesterone. Approximately 7% of testosterone can then be converted to a more potent metabolite, dihydrotestosterone (DHT) via 5α-reductase, and small amounts (approximately 0.5%) to oestrogen via P450 aromatase [24–26].

Testosterone binds with high affinity to the cytosolic or membrane AR and then regulates male sex development and maturation [25] as well as having crucial extra-gonadal effects including regulation of apoptosis via cleavage of procaspase 8 in VSMC [14], regulation of leucocyte migration and reactive oxygen species (ROS) generation [27], control of the nitric oxide (NO)–cGMP pathway [2] and improvement of insulin sensitivity [28]. Testosterone has also been used clinically in erectile dysfunction (ED), infertility, osteoporosis, to promote bone marrow stimulation and to stimulate penile enlargement and height growth [29,30]. In athletes, testosterone has been shown to enhance performance via muscle development, improved strength and endurance [31]. Moreover, androgens have been implicated to play a role in pathological processes when dysregulated [32].

Testosterone has been associated with cardiovascular pathology as evidenced by a higher male susceptibility to cardiovascular disease [19,33]. However, there is increasing evidence that low endogenous levels of testosterone may also be associated with cardiac dysfunction [34–36]. A reduction in total testosterone of 2.18 SD is associated with a 25% increased risk of cardiovascular mortality [37]. To date, the role of androgens in cardiovascular health and disease remains controversial.

*AR* is a single copy gene found on the X chromosome at Xq11-12 and mutations and polymorphisms in it are thought to be inversely proportional to the transcriptional response to testosterone [38]. To regulate target gene transcription, testosterone and DHT can bind to the AR in a DNA binding-dependent manner leading to new protein synthesis [25], or in a non-DNA binding-dependent manner that involves a rapid induction of secondary messengers to initiate cellular events, such as protein phosphorylation [39]. DHT is more biologically active than testosterone, which is associated with the 2-fold higher affinity for the AR and a reduction of 5-fold in the dissociation rate compared with testosterone [40].

## The classical DNA binding-dependent actions of the androgens

In the basal state, without ligand binding, the AR is located primarily in the cytoplasm where it associates with heat shock proteins (HSPs), which are thought to tether AR via cytoskeletal proteins and modulate AR conformation in preparation for efficient ligand binding [41,42]. The classical AR signalling pathway commonly referred to as ‘genomic’ or ‘canonical’ AR signalling involves androgens crossing the plasma membrane, entering the cytoplasm and binding to the AR, resulting in a dissociation of chaperone proteins, translocation of the complex to the nucleus where it dimerizes and binds to androgen response element (ARE) to modulate gene transcription and subsequently protein synthesis [40]. AR binding to specific ARE results in recruitment of histone acetyltransferase (HAT) enzymes and a number of essential co-regulators [41]. This facilitates binding of TATA-binding protein (TBP) followed by general transcription factors (GTF and RNA pol II) to begin transcription and to regulate the expression of androgen-regulated genes [41,43].

### Genomic action of the AR in vascular calcification

The relationship between testosterone and calcification, which is an important predictor of morbidity and mortality from cardiovascular disease, has been explored [44]. There is significant sexual dimorphism in the development of vascular calcification; males tend to have higher levels of calcium deposition, and this has been attributed as potentially secondary to the effects of the AR [45]. Recently, it was demonstrated that higher expression of AR occurs in calcified human aortic valve compared with controls. Treatment with androgens (testosterone or DHT) for 9 days resulted in up-regulation of AR expression in WT mice and also induced calcification, as shown by elevated calcium deposition and mRNA expression of *Alpl*, a marker of cellular mineralization, effects reduced in specific AR-ablated VSMC with a concurrent reduction in the mRNA expression of the osteogenic marker Osterix [46]. Other studies indicate the opposite, testosterone as an anti-calcification agent. Growth arrest-specific gene 6 (Gas6) is an important molecule regulating calcification of VSMC [47]. Gas6 is considered a pro-survival agent that reduces apoptosis, an essential process for VSMC calcification [48]. In VSMC, AR was found to directly bind to the ARE in the *Gas6* promoter region and to transactivate *Gas6*. This resulted in inhibition of inorganic phosphate (P_i_)-induced calcification of vascular cells. Restoration of Gas6 signalling induced by testosterone is mediated by phosphorylation of the phosphoinositide 3-kinase (PI3K)/protein kinase B (Akt) pathway, and an increase in anti-apoptotic Bcl2 family proteins. This effect is blunted by AR antagonists [49] and provides a mechanism behind the possible cardioprotective action of androgens, as suggested by the high levels of vascular calcification in men with hypogonadism [45]. Testosterone also regulates VSMC senescence via Gas6 activation. Angiotensin II (Ang II)-induced down-regulation of Gas6 in VSMC is restored by testosterone, which is followed by reduced expression and activity of MMP-2 and reduced Ang II-induced collagen synthesis effects not observed in the presence of Gas6 blockers and Axl-Fc and PI3K inhibitors. These results suggest a novel mechanism that involves Gas6/Axl and Akt in the protective effects of testosterone on vascular ageing [50].

### Genomic action of the AR in renal function, cardiac function and vasodilation

Functionally, active AR is also thought to be integral to the maintenance of normal cardiac and renal function. Cardiac and renal hypertrophy is common in Fabry disease, a condition caused by the deficiency of lysosomal enzyme α-galactosidase A [51]. The α-galactosidase A knockout mice, a model of Fabry disease, demonstrate increased mRNA and activity of AR in heart and kidneys, as indicated by increased expression of insulin-like growth factor 1, an androgen regulated gene, and reduced expression of transforming growth factor-β1, which is negatively regulated by AR. Castration and the consequent hypogonadism or AR-antagonism therapy results in a significant reduction in Akt phosphorylation and an improved phenotype in the heart and kidneys of Fabry disease model mice [52]. Echocardiography demonstrated improved heart-to-body weight ratios and left ventricle wall thickness and cardiac atrial natriuretic peptide mRNA levels in castrated mice. Kidney weight also remained at WT level in these mice [52].

To explore the role of the AR in cardiac growth, Ikeda et al. used 25-week-old AR knockout (ARKO) mice and age-matched wild-type male mice, which were treated with or without Ang II stimulation at a dose of 2mg/kg per day for 2 weeks [53]. The importance of the AR in cardiac development and function is highlighted by the phenotype of AR knockout animals, as ARKO mice have a significant reduction in cardiac hypertrophy induced by Ang II and heart-to-body weight ratio compared with WT, events associated with lower activation of extracellular signal-regulated kinases (ERKs) 1/2 and ERK5. In addition, impairment of left ventricle function and cardiac fibrosis induced by Ang II is reduced in ARKO mice [53].

Cross-talk between cytosolic and nuclear signalling pathways is involved in testosterone-induced cardiac hypertrophy. Glycogen synthase kinase 3 (GSK-3β) is considered an anti-hypertrophic factor in cardiac cells [54]. In cardiomyocytes, treatment with testosterone leads to phosphorylation of GSK-3β inhibitory site (Ser^9^), an increase in intracellular levels of calcium with consequent activation of calcineurin and nuclear factor of activated T cells (NFAT) and an increase in both cell size and [^3^H]-leucine incorporation, which suggest cardiomyocyte hypertrophy [55], suggesting that GSK-3β may be a pharmacological target to inhibit testosterone-induced cardiac hypertrophy.

Vasodilation, a well-described event associated with testosterone, seems to be, at least in part, regulated by AR activation. Hydrogen sulphide (H_2_S) is considered a prominent endothelium-derived hyperpolarizing factor that induces vasodilation via TRPV4 and large-conductance Ca^2+^-activated K channels [56]. Testosterone stimulation in thoracic aorta from male Wistar rat results in a concurrent increase in the production of H_2_S, and associated vasodilation, which is AR-dependent [57–59]. Interestingly high levels of H_2_S inhibit AR binding in human prostate cancer cells, suggesting a tissue-specific feedback loop, which may offer future treatment options for castration resistant prostate cancer [60]. Moreover, chronic stimulation with androgens (24 h) seems to have a direct effect in endothelial cells. DHT increases the levels of vascular endothelial growth factor and improves the proliferative, migratory and adhesive abilities of endothelial progenitor cells, events regulated by the RhoA/ROCK pathway [4].

Androgens also have a direct effect on Ang II type-2 receptor (AT2R) expression. In aortas isolated from male rats, AT2R mRNA and protein expression levels are lower than in females. The elevated level of AT2R mRNA and protein expression in endothelium-intact aorta from female rats can be reversed by DHT administration, an effect attenuated through co-administration of AR antagonist and not observed in the presence of an ERK1/2 inhibitor. Interestingly, DHT therapy in females did not alter AT2R expression in endothelium-denuded aorta. On the other hand, castration of male rats significantly elevated AT2R mRNA and protein expression, suggesting that independent of the sex, testosterone via AR has a direct effect on expression of the Ang II receptor [61].

### Genomic action of the AR in erectile function

ED provides another example of the clinical significance of the genomic effects of androgens. ED is considered a complex neurovascular phenomenon and hypogonadism is the most common risk factor for this, leading to insufficient arterial blood flow to the penis [62–65]. Castrated rats exhibit impaired erectile function and internal pudendal arteries from castrated animals demonstrate impairment in vasoconstrictor and vasodilator function, which is associated with hypotrophic vascular remodelling, decreased neuronal nitric oxide synthase (nNOS) and α-actin expression and increased collagen expression, p38 mitogen-activated protein kinases (p38) phosphorylation and caspase 3 cleavage [66]. In penile tissue, AR expression reduces in an age-dependent manner and also in a testosterone dose-dependent manner. The reduced AR expression may therefore play a role in the ED vascular phenotype [67].

**Figure 1:**
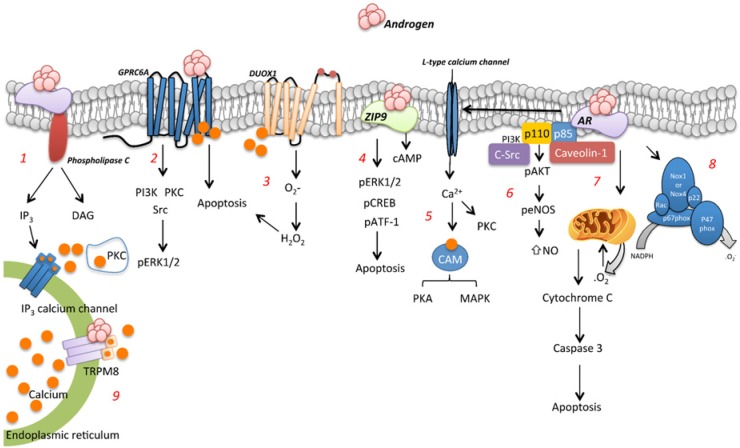
DNA binding-dependent signalling induced by androgens (1) The genomic AR signalling involves androgen crossing the plasma membrane, entering the cytoplasm, dissociation of chaperone proteins and binding to the AR. (2) Testosterone induced-ROS generation is followed by an increase in Nox1 and Nox4 mRNA levels and p47phox protein expression. (3) Gas6 signalling induced by testosterone is mediated by phosphorylation of the PI3K/Akt pathway, and an increase of anti-apoptotic Bcl2 family proteins. (4) Hypertrophy induced by testosterone involves recruitment of NFAT through calcineurin activation and GSK-3β inhibition. (5) Testosterone down-regulates the AT2R receptor via AR-mediated ERK1/2 activation. (6) Hypogonadism is shown to decrease nNOS and α-actin expression and increase p38 phosphorylation and caspase 3 cleavage. (7) Testosterone stimulation results in a concurrent increase in the production of H_2_S, and consequently vasodilation via TRPV4 and large-conductance Ca^2+^-activated K-channels.

Table 1 and Figure 1 summarise the genomic actions of androgens on the vasculature [4,46,49,50,52,53,55–59,61].

**Table 1:** Summary of the genomic effects of androgens

Androgen	Cell/Tissue	Effect	Reference
Testosterone DHT	VSMC mouse	↑ AR expression ↑ Calcium deposition ↑ mRNA expression *Alpl*	Zhu et al. (2016) [46]
Testosterone	Human aortic smooth muscle cells	Phosphorylation of PI3K/Akt Transactivation *Gas6*	Son et al. (2010) [49]
Testosterone	VSMC mouse	↓ Senescence ↓ Expression MMP-2 ↓ Ang II-induced collagen synthesis	Chen et al. (2016) [50]
Testosterone	α-galactosidase A knockout mice	↑ AR expression and mRNA ↓ Akt phosphorylation with castration	Shen et al. (2015) [52]
Testosterone	ARKO mice	↓ ERK 1/2 and ERK 5	Ikeda et al. (2005) [53]
Testosterone	Cardiomyocytes	Phosphorylation GSK-3β inhibitory site (Ser^9^) ↑ Intracellular calcium ↑ Calcineurin and NFAT ↑ Cell size	Duran et al. (2016) [55]
Testosterone	Thoracic aorta rat	↑ H_2_S production ↑ Vasodilation	Bucci et al. (2009) [57] Brancaleone et al. (2015) [58] Mustafa et al. (2011) [59]
DHT	Endothelial progenitor cells	↑ VEGF ↑ Proliferation, migration and adhesion of cells	Zhang et al. (2016) [4]
DHT	Rat aorta	↓ AT2R mRNA and protein expression ↑ ERK 1/2 activation	Mishra et al. (2016) [61]

## Non-DNA binding-dependent actions of the androgens

In the past, interactions with a nuclear sex hormone receptor followed by transcription factor activity were implicated as the principal molecular mechanism responsible for androgen activity. However, there is increasing evidence that androgens can also act via mechanisms independent of the ligand-dependent transactivation function of nuclear receptors [68]. This is known as ‘non-genomic’ signalling, which typically occurs within a short time frame [69].

**Figure 2:**
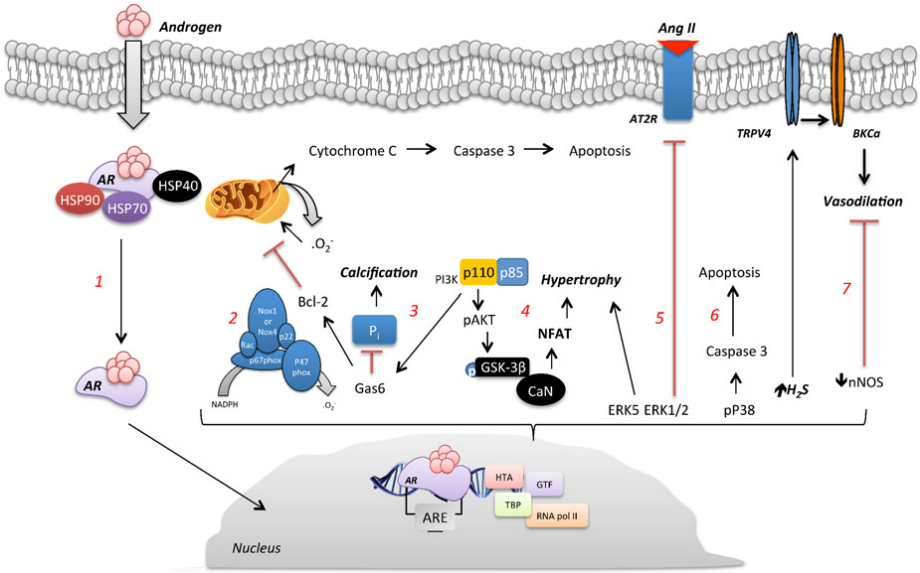
Non-DNA binding-dependent signalling induced by androgens (1) Testosterone via rapid response activates PLC, IP_3_ and DAG and initiates intracellular calcium release and PKC activation. (2) Via binding to GPRC6A, testosterone leads to ERK phosphorylation by mechanisms involving PI3K, PKC and Src. (3) GPRC6A mediates the non-genomic effects of testosterone on intracellular calcium mobilization and H_2_O_2_ through Duox1. (4) ZIP9 activation induced by testosterone is involved in testosterone induced ERK1/2, CREB and pATF-1 phosphorylation. (5) Via interaction with AR, androgens activate L-type calcium channels, which increase the intracellular levels of calcium, activate PKC, and via calmodulin activate PKA and MAPK pathways. (6) Activation of PI3k/Akt signalling and the direct interaction of AR with p85α/c-Src/caveolin1 are involved in testosterone-induced eNOS phosphorylation. (7) Testosterone increases mitochondrial-ROS generation and procaspase-8 and -3 activation in VSMC, an effect followed by reduction of O_2_ consumption, increased expression of death receptors and apoptosis. (8) Rapid generation of ROS induced by testosterone involves NAPH oxidase activation. (9) Androgen binding to TRPM8 is followed by an increase in TRPM8-induced increase in intracellular levels of Ca^2+^.

To be considered a non-genomic response, the androgen-induced response must occur in a time frame not long enough to allow gene transcription, normally seconds to minutes. The response should be observed even when the androgen is conjugated to molecules such as bovine serum albumin (BSA) that prevent it from entering into the cytoplasm. A third criterion requires that the non-genomic response should not be blunted by inhibitors of transcription and does not require a functional nucleus or transcription/translation machinery activation [39,69,70]. The non genomic actions of androgens in the vasculature are summarised in Figure 2 and Table 2.

**Table 2:** Summary of non-genomic effects of androgens on vascular tissue

Androgen	Cell/Tissue	Effect	Reference
Testosterone	Murine macrophages	↑ Intracellular calcium ↑ PLC Activation RAS/MEK/ERK MAPK pathways	Wunderlich et al. (2002) [75] Foradori et al. (2008) [39]
Testosterone	Cardiac myocytes	↑ Release Ca^2+^ from endoplasmic reticulum and mitochondria	Bennett et al. (2010) [41] Foradori et al. (2008) [39]
Testosterone	Male rat osteoblasts	↑ Cytosolic free calcium ↑ IP_3_ and DAG formation	Lieberherr et al. (1994) [74]
Testosterone	AEC	Rapid ↑ NO eNOS phosphorylation PI3K, cavceolin-1 and c-Src binding to AR AKT phosphorylation	Son et al. (2010) [49] Yu et al. (2010) [79]
Testosterone	VSMC	Inhibits PGF2α induced Ca^2+^ influx	Ma et al. (2009) [81]
Testosterone	Fingertip arterial pulse	↓ Arterial stiffness Acute vasodilation	Francomano et al. (2016) [82]
Testosterone	Prostate cancer cells	TRPM-8 induced ↑ intracellular Ca^2+^	Zhang et al. (2004) [89]
Synthetic R1881 Testosterone	HEK-293 cells transfected with GPRC6A	↑ ERK activity	Pi et al. (2010) [128]
Testosterone	VSMC rats	ROS generation ↑ Nox1 and Nox4 mRNA ↑p47phox protein VSMC migration	Chignalia et al. (2012) [129]
Testosterone	Epidermal keratinocytes	Rapid Ca^2+^ mobilization from endoplasmic reticulum ↑ H_2_O_2_ generation ↓ Mitochondrial membrane potential ↓ Apoptosis ↑ Duox1 stimulation ROS generation	Ko et al. (2014) [96]
Testosterone	93RS2 Sertoli cells	ERK1/2 phosphorylation CREB phosphorylation ATF-1 phosphorylation	Bulldan et al. (2016) [86]
Testosterone	Prostate and breast cancer cell lines	Activation of G proteins Up-regulation JNK gene expression ERK phosphorylation ↑ Pro-apoptotic Bax, caspase 3, cytochrome *c* proteins Apoptosis	Thomas et al. (2014) [87]
Testosterone	VSMC	Mitochondrial ROS generation Procaspase-8 and -3 activation ↓ O_2_ consumption Apoptosis	Lopes et al. (2014) [14]

### Calcium mobilization and vascular function

The most robust evidence that androgens induce cellular effects through non-genomic signalling is the rapid rise of intracellular calcium concentration [71,72]. Hypogonadism is associated with an increased risk of osteopenia and osteoporosis, an effect normalized by testosterone replacement [73]. In male rat osteoblasts, low concentrations of testosterone, 10 pm/l–1 nM/l increase cytosolic free calcium and membrane phospholipid metabolism in a very rapid time (5–60 s), an effect followed by an increase in the cellular content of inositol 1,4,5-trisphosphate (IP_3_) and diacylglycerol (DAG) formation, events not observed in female rat osteoclasts, which suggest that the rapid effect induced by testosterone is sex dependent. Interestingly, androgen-induced increases in intracellular levels of calcium are also observed in testosterone conjugated with BSA, suggesting a response that involves membrane embedded or associated receptors or binding proteins [74]. Androgens/AR can also activate L-type calcium channels, which increase the intracellular levels of calcium, activate protein kinase C (PKC) and via calmodulin activate protein kinase A (PKA) and MAPK pathways [39].

Non-genomic Ca^2+^ mobilization by androgens was also observed in murine macrophages. In macrophages, testosterone increases intracellular levels of Ca^2+^ [75]. During this process, androgen interacts with membrane-associated AR, modulates G-protein activity and subsequently activates phospholipase C (PLC). This results in the rapid release of intracellular calcium stores from the sarcoplasmic reticulum and consequently activation of the RAS/MEK/ERK MAPK pathway [39]. Likewise through activation of plasma membrane AR associated with GPCR signalling in cardiac myocytes, stimulation with testosterone induces the release of Ca^2+^ from internal stores, such as endoplasmic reticulum and mitochondria [39,41].

Acute testosterone-induced non-genomic vasodilatation is mediated in part via endothelium-derived NO [76,77]. Aortic endothelial cells (AEC) stimulated with testosterone or non-permeable testosterone-BSA at physiological concentrations (1–100 nm) present rapid (15–30 min) increases in NO level in AEC; testosterone also induces endothelial nitric oxide synthase (eNOS) phosphorylation (Ser^1177^) without changing the total protein level. Activation of eNOS occurs via PI3K, caveolin-1 and c-proto-oncogene tyrosine-protein kinase (Src) binding to AR and consequently phosphorylation of AKT. AR and s-Src mediate testosterone-induced rapid eNOS phosphorylation, since pre-treatment with nilutamide or PP2, an AR and s-Src antagonist respectively, abolishes the testosterone responses. Transcriptional inhibitor, actinomycin D does not affect testosterone-induced increase in NO, which excludes the classical genomic actions [78,79]. Anastrozole or other oestrogen receptor antagonists do not interfere in NO generation induced by testosterone, suggesting that this is not an event associated with the aromatization of testosterone to oestradiol [80]. In addition, testosterone at physiological concentrations inhibits PGF2α-induced Ca^2+^ fluxes by a non-genomic mechanism in VSMC [81], which may contribute to testosterone-induced vasodilatation.

Vasodilatation not associated with DNA-binding induced by testosterone is also observed in humans. A recent paper demonstrated that administration of transdermal testosterone in men with hypogonadism and severe hypotestosteronaemia causes an acute vasodilation and improves arterial stiffness by non-genomic mechanisms, although interestingly, the improvement is also evident after 96 h of treatment, which would suggest a combination of genomic and non-genomic effects to reach the same response [82].

The molecular mechanisms underlying non-genomic actions include not only the translocation of the AR to the cell surface membrane [25] as many types of cells that demonstrate a rapid androgen response do not express the classic nuclear AR or are not blocked by AR antagonists, suggesting that in addition to the traditional AR, other proteins are capable of binding androgens and activating signal transduction cascades [39]. In addition to AR, androgens can also bind to lipids of the plasmatic membrane, promoting direct modification of ion channels [69], activation of GPRC6A [83–85] and interaction with ZIP9, a Zn^2+^ transporter from the family of the zinc-regulated transporter (ZRT), iron-regulated transporter (IRT)-like proteins [86,87].

An important example of testosterone-induced signal transduction cascades not dependent of binding to AR is the androgen binding to transient receptor potential cation channel subfamily M member 8 (TRPM8). TRPM8 plays an important role in the pathophysiology of prostate cancer and is considered an ionotropic testosterone receptor [88]. In prostate cancer LNCaP cells, TRPM8 acts as a Ca^2+^-permeable channel and is expressed in the endoplasmic reticulum and plasma membrane. siRNA or inhibition of TRPM8 is associated with apoptosis of LNCaP cells. Increased levels of testosterone are followed by a greater TRPM8-induced increase in intracellular levels of Ca^2+^ [89]. Picomolar concentrations of testosterone elicited Ca^2+^ responses and channel currents, and those were inhibited in the presence of a TRPM8 antagonist [90]. Considering that TRPM8 has an important role in cell survival and that TRPM8 is modulated by androgens, it might be predicted that anti-androgen therapy decreases the percentage of LNCaP viable cells via reduction of TRPM8.

GPRC6A is expressed in many tissues including bone marrow stromal cells, monocytes, prostate cancer cells, skeletal muscle cells, vascular smooth muscle and endothelial cells and Leydig cells [83,91]. Synthetic androgen R1881 and testosterone alone or conjugated with BSA, in a calcium dependent manner, rapidly stimulate ERK activity in HEK-293 cells (which lack both the AR and GPRC6A receptor) transfected with GPRC6A, but not in the non-transfected HEK-293 controls. This effect is reversed by an MAPK inhibitor, PI3K inhibitor, Src inhibitor and PKC inhibitor; flutamide has no effect on testosterone-stimulated GPRC6A activation of p-ERK. Interestingly, R1881 does not stimulate ARE-luciferase activity in HEK-293 cells expressing only GPRC6A, but does stimulate HEK-293 cells transfected with AR, suggesting activation of nuclear receptor signalling. *In vivo*, testosterone-induced ERK phosphorylation in the bone marrow and testes is markedly attenuated in GPRC6A^−/−^ mice, demonstrating that GPRC6A is a non-classical receptor for which androgens induce ERK activation both *in vitro* and *in vivo* [92].

### ROS generation and apoptosis

Testosterone has been implicated in hypertension-induced vascular remodelling, an event associated with ROS generation. ROS have been recognized as important messengers in cell signalling [93]. Testosterone induces ROS generation in VSMC isolated from normotensive (Wistar Kyoto, WKY) and hypertensive (spontaneously hypertensive rat, SHR) rats, an effect followed by an increase in nicotinamide adenine dinucleotide phosphate oxidase (Nox)1 and Nox4 mRNA levels and p47phox protein expression and VSMC migration. Curiously, rapid ROS generation in SHRSP is not inhibited by flutamide or actinomycin D, indicating a non-genomic effect. The complexity of the assessment of the genomic and non-genomic effects of testosterone in the vasculature is highlighted in this study as testosterone augmented ROS formation after 2 h was blocked by flutamide and actinomycin D, suggesting a genomic pathway and demonstrating that testosterone has the potential to act in multiple different ways in the same cells [94].

In addition to Nox1 and Nox4, the aforementioned isoforms, the Nox family includes five others isoenzymes (gp91 phox, renamed Nox2, Nox3, Nox5, Duox1 and Duox2) [95]. In epidermal keratinocytes, testosterone stimulation results in rapid and transient Ca^2+^ mobilization from the endoplasmic reticulum within 1 min, an effect reduced by transfection of GPRC6A siRNA and followed by an increase in H_2_O_2_ generation. Testosterone-induced H_2_O_2_ generation is not reverted by flutamide but not observed in the presence of DPI, an Nox inhibitor. ROS generation is not observed in Duox1-silenced or GPRC6A-silenced keratinocytes. Stimulation of keratinocytes with testosterone results in decreased mitochondrial membrane potential and apoptosis, an event also regulated by GPRC6A-dependent Ca^2+^ mobilization. Together, these results suggest that GPRC6A-dependent Ca^2+^ mobilization stimulates the activity of Duox1, leading to ROS generation and apoptosis [96]. The pro-apoptotic effect of testosterone is also observed in VSMC. Testosterone increases mitochondrial-ROS generation and procaspase-8 and -3 activation in VSMC, an effect followed by reduction of O_2_ consumption, increased expression of death receptors and apoptosis [14]. It has not yet been investigated whether GPRC6A or Duox-1 are also involved in testosterone-induced ROS generation and apoptosis in VSMC, which would be a new mechanism by which testosterone influences vascular function and may play a role in cardiovascular diseases. Together, the previous results suggest that it is possible that the effects in the vascular system induced by androgens are mediated by signalling cascades activated by oxidative stress, which highlights ROS as an important target particularly in patients with augmented testosterone levels.

It has been demonstrated that GPRC6A is not only an androgen target but also an androgen regulator. GPRC6A knockout mice display feminization of the external genitals, characterized by reduction in genitoanal distance as well as testicular size, which is followed by reduced levels of testosterone, but no difference in AR levels. Interestingly, oestradiol concentrations are significantly higher in male GPRC6A^−/−^ mice compared with wild-type littermates, an event associated with the increase in aromatase expression, a protein responsible for catalysing the oestrogen biosynthesis from androgens. GPRC6A is highly expressed in the kidney [97] and GPRC6A^−/−^ mice present an increase in urinary calcium and phosphate excretion and also excretion of β2-microglobulin. In addition, liver from GPRC6A^−/−^ mice exhibits histological markers of hepatic steatosis [98]. Considering that GPRC6A is involved in the rapid effects of testosterone and that GPRC6A has multiple functions, it is possible that many important non-genomic effects induced by testosterone are still unknown.

In addition to GPRC6A, non-DNA binding-dependent actions of testosterone can also be associated with androgen binding to ZIP9, a membrane-integrated receptor [86]. In this case, ZIP9 would perform dual functions as a membrane AR and zinc transporter. In 93RS2 Sertoli cells, a cell line that does not express AR, testosterone (10 nM) induces ERK1/2 phosphorylation. Similar effects were also observed in cAMP response element-binding protein (CREB) and activating transcription factor 1 (ATF-1) phosphorylation, effects suppressed by ZIP9 siRNA, indicating that ZIP9 is involved in the testosterone-induced signalling pathway [86].

As previously mentioned, testosterone induces apoptosis in different types of cells, including VSMC [14,89,96], an event where ZIP9 seems to play an important role. In cancer cells, testosterone stimulation induces activation of G proteins, up-regulation of JNK gene expression, ERK phosphorylation and increased expression of pro-apoptotic Bax, caspase 3 and cytochrome *c* proteins, culminating in apoptosis. Transfection of ZIP9 siRNA is accompanied by a complete loss of testosterone-induced apoptosis [87]. It has not yet been investigated whether this novel steroid signalling pathway is initiated through the zinc transporter ZIP9 in VSMC also.

## Implications for androgen therapy in cardiovascular disease

The use of androgens leads to effects that range from protective to deleterious [99], resulting in an ongoing debate regarding the clinical benefits and long-term risks of testosterone therapy. As previously mentioned, genomic and non-genomic effects of testosterone can result in different responses, which may help us to understand the divergent outcomes of testosterone therapy in the cardiovascular system.

Men with low testosterone have a high prevalence of cardiovascular disease and metabolic syndrome [100–102] and testosterone therapy in these individuals has been associated with reduced obesity, fat mass, waist circumference and mortality as well as improved glycaemic control and overall cardiometabolic status compared with placebo [103]. On the other hand, testosterone supplements are known to increase haematocrit levels and reduce HDL (high-density lipoprotein) cholesterol levels and have been implicated in cases of cardiovascular morbidity and mortality [104]. Despite this, a systematic review and meta-analysis evaluating the cardiovascular effects of testosterone supplementation in 3016 men who were supplemented with testosterone and 2448 placebo-treated men found no causal role between testosterone supplementation and cardiovascular events [105]. The results are so divergent that even the route of administration of testosterone may be associated with differing cardiovascular risk [106].

A prospective study with 11,606 men, aged 40–79 years, found that testosterone baseline levels are inversely related to mortality due to all causes, cardiovascular diseases and cancer [34]. All-cause mortality is increased in hypogonadal men with Type 2 diabetes and testosterone therapy reduces mortality to 8.4% compared with 19.2% in the untreated group [107]. Corroborating these results, a large multi-centre, randomized, double-blind, placebo-controlled study undertaken in eight European countries in men with Type 2 diabetes and/or the metabolic syndrome showed that testosterone replacement therapy (TRT) improves cardiovascular risk factors in men, including body fat composition, cholesterol, insulin resistance and sexual function [28].

In humans, low testosterone levels are associated with endothelial dysfunction [108], which can be reverted by testosterone therapy. The vascular function of male patients as examined by the vasomotor function of the brachial artery and intima-media thickness of the carotid artery demonstrated that low levels of testosterone are associated with endothelial dysfunction, independent of body mass index, presence of diabetes, hyperlipidaemia or hypertension and age [109].

However, protective effects in the heart are also observed with testosterone therapy. Haemodynamic parameters in patients and animal models of heart failure are improved by testosterone therapy, mainly via increased coronary blood flow through vasodilation, reduction in peripheral vascular resistance and via direct effects in the cardiac tissue such as inhibition of cardiac cAMP phosphodiesterases [110,111]. These potentially protective effects have been considered in a recent clinical study by Cheetham et al. [112], which compared a group of 8808 men who had received TRT and 35,527 men who had never received TRT. The primary outcome of the study was an amalgamation of incidence of acute myocardial infarction, coronary revascularization, unstable angina, stroke, transient ischaemic attack and sudden cardiac death. The rates of cardiovascular disease, as determined by this amalgamation of information were 23.9 in the no-TRT group versus 16.9 per 1000 in the TRT groups, demonstrating that testosterone supplementation in men with androgen deficiency results in a reduced risk of adverse cardiovascular outcomes [112].

Low-density lipoprotein, total cholesterol and triglycerides are reduced by testosterone therapy; in addition, testosterone replacement increases high-density lipoprotein (HDL) and inhibits fatty streak formation, suggesting a protective effect against atherosclerosis [113–115]. Similarly, some studies including 4-year follow-up study found that the degree of atherosclerosis progression is inversely associated with testosterone levels [116,117].

Despite the results mentioned above, androgens may have a deleterious influence in the cardiovascular system via an increase in blood pressure and renal dysfunction. Increased renal vascular resistance and ROS generation in male elderly SHR are prevented by orchiectomy [118,119]. Likewise infusion of DHT in animals is associated with an increase in blood pressure, increase in sodium and water reabsorption and also ROS generation, an effect reversed by 6 weeks of treatment with the SOD mimetic tempol [120,121]. As mentioned before testosterone also induces ROS generation and apoptosis of VSMC [14], increases the level of important sources of ROS and NADPH oxidase [94] and also decreases the expression of antioxidant enzymes [122], which suggests that detrimental effects induced by testosterone can involve an imbalance between pro- and-antioxidant systems, leading to oxidative stress.

Deleterious effects of testosterone are also observed in females. Increased testosterone levels in women with polycystic ovary syndrome are associated with cardiovascular and metabolic disease. These patients have a 2-fold-increased risk for arterial disease independent of body mass index, hypertension and diabetes status [123,124]. In addition, women with cardiovascular disease have higher levels of free androgen compared with controls [125].

Many studies have demonstrated that an equilibrium in testosterone levels is essential for the appropriate function of many signalling pathways in different organs and tissues (Figures 1 and 2). However, the protective/deleterious effect induced by testosterone therapy remains controversial. Differential effects are possibly due to activation of distinct sets of signalling pathways. The European Male Ageing Study (EMAS) is a prospective multi-centre approach that followed up 1887 men over a median of 4.3 years [126]. They investigated the *AR* gene exon 1 CAG repeat length of these men, as this may be associated with androgen action [127]. In individuals with longer repeats, an intact gonadal axis compensated for this adverse genetic background, meaning that affected individuals with normal hormone biochemistry did not have an increased risk of cardiovascular or other medical conditions, suggesting that the effect of testosterone may be mediated through non-genomic actions in these individuals [126]. It is clear that the improved understanding of genomic and non-genomic effects of testosterone will therefore lead to the potential for development of novel therapeutic targets for patients with cardiovascular disease.

## Conclusions

It is clear that genomic and non-genomic effects induced by androgens have implications for the development of cardiovascular disease. These effects range from protective, such as reduced fat mass, to deleterious including activation of pro-apoptotic and pro-oxidant signalling pathways. Accordingly, a consensus has yet to be reached regarding the effects of testosterone on the cardiovascular system. Considering that even testosterone-induced genomic and non-genomic responses could lead to divergent outcomes such as rapid improvement of vascular function via release of NO and hydrogen sulphide or chronic vascular dysfunction associated with calcification, the improved understanding of the mechanisms by which testosterone induces acute or chronic responses could be crucial to comprehend the discrepant outcomes induced by androgens. Given that cardiovascular disease remains a major cause of human death, particularly in males and post-menopausal women, further translational research in the field of genomic and non-genomic effects of testosterone may lead to novel therapeutic targets for patients in the future.

## Abbreviations

AEC: aortic endothelial cells
Akt: protein kinase B
Ang II: angiotensin II
AR: androgen receptor
ARE: androgen response element
ARKO: androgen receptor knockout
ATF-1: activating transcription factor 1
AT2R: angiotensin II type-2 receptor
Bax: bcl-2-like protein 4
Bcl2: B cell lymphoma 2
BSA: bovine serum albumin
cAMP: cyclic adenosine monophosphate
CREB: cAMP response element-binding protein
DAG: diacylglycerol
DBD: DNA-binding domain
DHEA: dehydroepiandrosterone
DHT: dihydrotestosterone
DNA: deoxyribonucleic acid
DPI: diphenyleneiodonium
Duox1: L dual oxidase 1
ED: erectile dysfunction
eNOS: endothelial nitric oxide synthase
ERK: extracellular signal-regulated kinases
ERK 1/2: extracellular signal-regulated kinases 1/2
GPCR: G protein coupled receptor
GSK-3β: glycogen synthase kinase 3
H_2_O_2_: hydrogen peroxide
H_2_S: hydrogen sulfide
HAT: histone acetyltransferase
HDL: high-density lipoprotein
HSP: heat shock protein
IP_3_: inositol trisphosphate
IRT: iron-regulated transporter
JNK: c-Jun N-terminal kinases
LBD: ligand-binding domain
LNCaP: cell line derived from androgen sensitive human prostate adenocarcinoma cells
MAPK: mitogen activated protein kinase
MEK: mitogen activated protein kinase kinase
MMP-2: matrix metalloproteinase 2
mRNA: messenger RNA
NADPH: nicotinamide adenine dinucleotide phosphate
NFAT: nuclear factor of activated T cells
nNOS: neuronal nitric oxide synthase
NO: nitric oxide
Nox: nicotinamide adenine dinucleotide phosphate oxidase
O_2_^−^: superoxide anion
p38: p38 mitogen-activated protein kinases
P_i_: inorganic phosphate
PGF2alpha: prostaglandin F2 alpha
PI3K: phosphoinositide 3-kinase
PKA: protein kinase A
PKC: protein kinase C
PLC: phospholipase C
RhoA: Ras homolog gene family member A
RNA: ribonucleic acid
ROCK: Rho associated kinase
ROS: reactive oxygen species
SD: standard deviation
SHR: spontaneously hypertensive rat
SHRSP: spontaneously hypertensive stroke prone
siRNA: small interfering RNA
Src: proto-oncogene tyrosine-protein kinase
TBP: TATA-binding protein
TRPM8: transient receptor potential cation channel subfamily M member 8
TRPV4: transient receptor potential cation channel subfamily V member 4
TRT: testosterone replacement therapy
VSMC: vascular smooth muscle cell
WT: wild type
WKY: Wistar Kyoto
ZIP9: zinc transporter protein 9
ZRT: zinc-regulated transporter
